# Anti-Aβ single-chain variable fragment antibodies restore memory acquisition in a *Drosophila* model of Alzheimer’s disease

**DOI:** 10.1038/s41598-017-11594-2

**Published:** 2017-09-12

**Authors:** Alfonso Martin-Peña, Diego E. Rincon-Limas, Pedro Fernandez-Funez

**Affiliations:** 1 Department of Neurology, McKnight Brain Institute, University of Florida, Gainesville, FL USA; 2 Center for Smell and Taste, University of Florida, Gainesville, FL USA; 3 Department of Neuroscience and Center for Translational Research on Neurodegenerative Diseases, Genetics Institute, University of Florida, Gainesville, FL USA; 4 Department of Biomedical Sciences, University of Minnesota Medical School, Duluth Campus, Duluth, MN USA

## Abstract

Alzheimer’s disease (AD) is a prevalent neurodegenerative disorder triggered by the accumulation of soluble assemblies of the amyloid-β42 (Aβ42) peptide. Despite remarkable advances in understanding the pathogenesis of AD, the development of palliative therapies is still lacking. Engineered anti-Aβ42 antibodies are a promising strategy to stall the progression of the disease. Single-chain variable fragment (scFv) antibodies increase brain penetration and offer flexible options for delivery while maintaining the epitope targeting of full antibodies. Here, we examined the ability of two anti-Aβ scFv antibodies targeting the N-terminal (scFv9) and C-terminal (scFv42.2) regions of Aβ42 to suppress the progressive memory decline induced by extracellular deposition of Aβ42 in *Drosophila*. Using olfactory classical conditioning, we observe that both scFv antibodies significantly improve memory performance in flies expressing Aβ42 in the mushroom body neurons, which are intimately involved in the coding and storage of olfactory memories. The scFvs effectively restore memory at all ages, from one-day post-eclosion to thirty-day-old flies, proving their ability to prevent the toxicity of different pathogenic assemblies. These data support the application of this paradigm of Aβ42-induced memory loss in *Drosophila* to investigate the protective activity of Aβ42–binding agents in an AD-relevant functional assay.

## Introduction

Alzheimer’s disease (AD) is the most common dementia with a prevalence of 11% among those older than 65^1^. For this age group, the risk of AD doubles every five years and one third of the population above 85 is affected^2^, highlighting its profound personal, medical, and social impact. AD is pathologically characterized by the accumulation of hyperphosphorylated tau in intracellular neurofibrillary tangles and the deposition of amyloid-β1-42 (Aβ42), a proteolytic product of the amyloid precursor protein (APP), in extracellular plaques^1^. Recent adjustments to the original amyloid hypothesis pose that soluble pre-amyloid structures, which include oligomers and protofibrils with varying degrees of β-sheet structure, are the most toxic Aβ42 species^3^. However, insoluble/fibrillar Aβ42 may still play relevant roles, including the release of small fragments that can seed Aβ42 oligomers^3, 4^. It is likely, then, that both soluble and insoluble Aβ42 assemblies contribute to disease and, therefore, therapeutic strategies should simultaneously tackle multiple Aβ42-based targets to achieve higher efficiency.

Immunotherapy is a promising approach for targeting and neutralizing Aβ42 neurotoxicity by directing antibodies against different Aβ42 domains, conformations, or assemblies. Passive immunotherapy via administration of humanized anti-Aβ42 antibodies has revealed promising results in preclinical studies^5, 6^. However, several clinical trials using passive immunization have recently reported disappointing results in symptomatic patients with mild cognitive impairment (MCI) and presymptomatic patients with high plaque load^7–10^. These studies concluded that two main factors constrain the efficiency of anti-Aβ42 immunotherapies: (1) neuronal loss is too advanced in symptomatic cohorts to significantly protect cognitive function and/or (2) the amount of full antibody entering the brain and binding Aβ42 in its primary target regions are low. Despite the lack of success in previous clinical studies, additional strategies employing immunotherapy still have the potential to combat Aβ42 neurotoxicity. In general, ongoing immunotherapy strategies are supported by robust preclinical results, the relative safety of passive immunotherapy, and the rationale that antibodies will bind Aβ42 and either promote its degradation or block its toxicity. One avenue for improving the performance of anti-Aβ42 antibodies is by exploiting the advantages of smaller antibody fragments through antibody engineering techniques. Single-chain variable fragment (scFv) antibodies are engineered antibodies comprised of the variable regions of the heavy and light chains connected by a short linker. ScFvs are easily delivered to the brain due to their low molecular weight (~30 kDa) and can be administered via injection of purified antibodies or introduced in small viral vectors^11^. Two anti-Aβ42 scFv antibodies targeting the N-terminal (Aβ1-16; scFv9) or C-terminal (Aβx-42; scFv42.2) regions of Aβ42 reduce plaque load in the CRND8 mouse model of AD that has no overt neurodegeneration^12^. Expression of the same two scFv antibodies protect against eye toxicity, neuronal death, dendritic degeneration, and locomotor dysfunction in a *Drosophila* model of human Aβ42 neurotoxicity^13^. In our previous work, we used locomotor dysfunction as a surrogate assay for monitoring neuronal activity over time. However, we were unable to provide critical functional evidence in an AD-relevant behavioral assay until this present study.

*Drosophila* has emerged as a model ideally suited to investigate the mechanisms of learning and memory at the molecular, cellular, and behavioral levels. Research over the last 30 years has uncovered significant similarities between *Drosophila* and mammals in the anatomical organization of the olfactory system and the molecular pathways underlying memory formation^14^. In *Drosophila*, the mushroom bodies (MB) are composed of approximately 2,000 cholinergic neurons in each side of the brain that constitute a major site for the formation and storage of olfactory memories^15–17^. Interestingly, ubiquitous expression of tau in the *Drosophila* brain selectively affects MB neurons, consistent with the neuron-specific pathology of AD^18, 19^. Olfactory classical conditioning and MB neurons constitute an ideal model to functionally analyze the efficiency of new therapeutic agents against the neurodegenerative effects of human amyloids. Fruit flies are also excellent to model human proteinopathies, including AD^20^. *Drosophila* models of AD overexpressing tau, APP/APPL (APP-like, the *Drosophila* orthologue of APP), or Aβ42 replicate relevant features of AD, including memory impairment^18, 19, 21–24^. In particular, pan-neuronal expression of Aβ42 or Aβ40 induces memory deficits in 6 day-old flies, whereas locomotor dysfunction is not observed until 20 days of age^24^, suggesting a higher sensitivity of the memory system to Aβ toxicity. Also, expression of Aβ42 carrying the Arctic mutation (E22G) induces more prominent memory loss than wild-type Aβ42, indicating that *Drosophila* learning and memory assays are particularly sensitive to clinically-relevant Aβ42 variants^25^. This memory loss paradigm has also contributed to elucidate new modifiers and pathways that interfere with Aβ42 neurotoxicity, which include zinc transporters^26^ and the epidermal growth factor^27^ and PI3K^28^ signaling pathways.

Here, we developed a sensitive behavioral assay for monitoring age-dependent memory decline in *Drosophila* and found that Aβ42 expression in MB neurons triggers a progressive impairment in memory formation for up to 30 days. We then proved that two known anti-Aβ42 scFv antibodies, scFv9 and scFv42.2, significantly increase memory performance in young and old flies expressing Aβ42, reaching similar levels to those of control flies. Together, these results support the physiological significance of this *Drosophila* paradigm of Aβ42-induced memory loss in examining the protective activity and therapeutic potential of Aβ42–binding agents.

## Results

### Aβ42 expression in MB neurons induces progressive memory impairment

**Figure 1:**
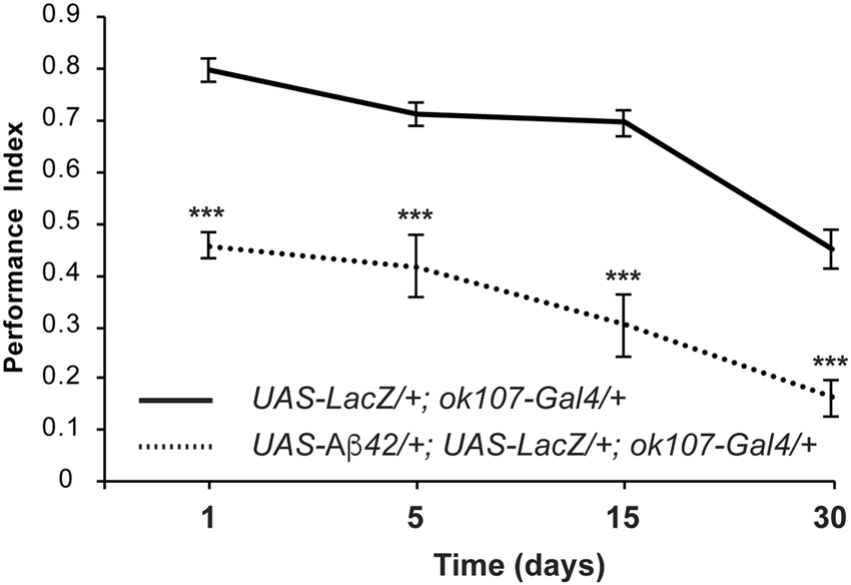
Memory acquisition is impaired in *Drosophila* expressing Aβ42. Flies were trained at days 1 (**a**), 5 (**b**), 15 (**c**), or 30 (**d**) post-eclosion using olfactory classical conditioning and tested immediately after training. Memory performance index is shown for control flies (*UAS-LacZ*/+; *ok107-Gal4*/+) and flies expressing Aβ42 in MB neurons (*UAS-Aβ42*/+; *UAS-LacZ*/+; *ok107-Gal4*/+). Control flies show physiological memory decay through aging (Tukey’s comparison test: day 1 vs day 5, *p* = 0.0275; day 5 vs day 15, *p* = 0.9873; day 15 vs day 30, *p* < 0.0001; day 1 vs day 30, *p* < 0.0001; day 5 vs day 30, *p* < 0.0001). Flies expressing Aβ42 exhibit lower memory values (Tukey’s comparison test: day 1 vs day 5, *p* = 0.7981; day 5 vs day 15, *p* = 0.6533; day 15 vs day 30, *p* = 0.3108; day 1 vs day 30, *p* = 0.0065; day 5 vs day 30, *p* = 0.0419). Flies expressing Aβ42 show lower memory index than control flies at each time point (t-test comparison: day 1, *p* < 0.0001; day 5, *p* < 0.0001; day 15, *p* < 0.0001; day 30, *p* < 0.0001). Error bars indicate SEM; n = 10 per group; ***p < 0.001.

As AD is characterized by progressive memory loss, we devised a *Drosophila* model of AD that exhibits dramatic memory impairment after olfactory classical conditioning. This model introduces several important novelties, including the use of a robust *Aβ*42 construct^29^, directed expression of Aβ42 to MB neurons, and extended testing of memory performance for up to 30 days to mimic the unrelenting progression of AD in older patients. We first compared memory performance of control flies (*UAS-LacZ*/+; *ok107-Gal4*/+) and flies expressing Aβ42 in MB (*UAS-Aβ42*/+; *UAS-LacZ*/+; *ok107-Gal4*/+) at days 1, 5, 15, and 30 post-eclosion. Control flies display high levels of memory acquisition at day 1 that gradually decay as flies age. These flies exhibit a slight but significant loss of memory from day 1 to day 5 (*p* = 0.0275). This is followed by a steady stage with no significant memory loss between days 5 and 15 (Fig. 1; *p* = 0.9873). This period of stability at the functional level correlates with periods of synaptic stability in other neuronal centers of the *Drosophila* brain^30–32^. From day 15 to 30, however, we observe a significant decline in memory performance (Fig. 1; *p* < 0.0001). This age-dependent memory decline has been previously described in flies^33^ and is a conserved trait^34^ due to impairment in the general physiology of neurons underlying memory loss and other behavioral changes^30, 31^.

Flies expressing Aβ42, though, perform at significantly lower levels than control flies at all ages tested (Fig. 1; *p* < 0.0001). The progression of memory loss in these flies is initially slow, with no significant differences between days 1 and 15 post-eclosion (Fig. 1; *p* = 0.1948), partly due to the dramatic memory impairment already present in one-day-old flies. Despite the slow progression, the memory deficits between days 1, 5, and 15 are significant compared to the memory levels of control flies at the same ages (Fig. 1; *p* < 0.0001). Additionally, memory performance is significantly impaired in thirty-day-old flies expressing Aβ42 compared to that of younger flies (Fig. 1). Moreover, flies expressing Aβ42 perform significantly lower than control flies at day 30 (Fig. 1; *p* < 0.0001). Despite this poor performance, the memory level in flies expressing Aβ42 is statistically different from zero (Wilcoxon test; *p* = 0.0142), suggesting that they still form weak memories. Together, these results support the use of our *Drosophila* paradigm of Aβ42-associated memory loss over 30 days with a significant sensitivity to discriminate between the natural memory loss in control flies and that related to Aβ42-neurotoxicity. These phenotypic differences in memory acquisition therefore allow evaluating the protective activity of antibodies and other agents.

### Aβ42 expression in MB does not impair stimuli perception

Since memory performance is significantly impaired in flies expressing Aβ42 in MB neurons, we asked whether these flies properly perceived the presented stimuli, which is a prerequisite to form memories. Hence, it is important to eliminate the possibility that these deficits are due to impairments in sensory perception. We then evaluated the avoidance index of control flies and flies expressing Aβ42 in MB against a 90 V electric shock (Table 1) and the two odors, octanol (Table 2) and benzaldehyde (Table 3). Overall, we report no significant differences between control and flies expressing Aβ42 in shock or odor perception at any time point (Tables 1–3). But, flies expressing Aβ42 exhibit a lower sensitivity to the two odors at days 5 and 30, although the differences with controls are not significant (Tables 2–3). Thus, the differences in memory performance described above (Fig. 1) are mainly due to deficits in memory formation.

**Table 1:** Electric shock avoidance of flies expressing Aβ42 over time.

90 V Shock Avoidance	*1-UAS-LacZ*/+; *ok107-Gal4*/+	*2-UAS-Aβ42*/+; *UAS-LacZ*/+; *ok107-Gal4*/+	*p-value*
1 day	0.7214 ± 0.0362	0.7342 ± 0.0389	*0*.*9999*
5 days	0.6744 ± 0.0512	0.7611 ± 0.0368	*0*.*9106*
15 days	0.6229 ± 0.0348	0.6029 ± 0.0346	*0*.*9999*
30 days	0.4212 ± 0.0452	0.3765 ± 0.0531	*0*.*9998*

Avoidance to an electric shock of 90 V for control flies (#1) and flies expressing Aβ42 (#2) and their *p*-value for statistical significance.

**Table 2:** Odor avoidance for 3-octanol of flies expressing Aβ42 over time.

3-Octanol Avoidance	1	2	*p-value*
1 day	0.7939 ± 0.0505	0.7028 ± 0.0315	*0*.*8531*
5 days	0.8116 ± 0.0121	0.5096 ± 0.1088	*0*.*0616*
15 days	0.4048 ± 0.0710	0.3438 ± 0.0568	*0*.*9999*
30 days	0.3624 ± 0.0793	0.3774 ± 0.0871	*0*.*3698*

Avoidance to the odor 3-octanol for control flies (#1) and flies expressing Aβ42 (#2) and their *p*-value for statistical significance. Complete genotypes: 1-*UAS-LacZ*/+; *ok107-Gal4*/+ and 2-*UAS-Aβ42*/+; *UAS-LacZ*/+; *ok107-Gal4*/+.

**Table 3:** Odor avoidance for benzaldehyde of flies expressing Aβ42 over time.

Benzaldehyde Avoidance	1	2	*p-value*
1 day	0.4338 ± 0.0485	0.3145 ± 0.0425	*0*.*8395*
5 days	0.3944 ± 0.0805	0.1420 ± 0.1144	*0*.*5339*
15 days	0.2258 ± 0.0480	0.1723 ± 0.0343	*0*.*9999*
30 days	0.2759 ± 0.0932	0.0892 ± 0.0497	*0*.*2403*

Avoidance to the odor benzaldehyde for control flies (#1) and flies expressing Aβ42 (#2) and their *p*-value for statistical significance. Complete genotypes: 1-*UAS-LacZ*/+; *ok107-Gal4*/+ and 2-*UAS-Aβ42*/+; *UAS-LacZ*/+; *ok107-Gal4*/+.

### Exogenous expression of scFvs do not alter memory formation in *Drosophila*

**Figure 2:**
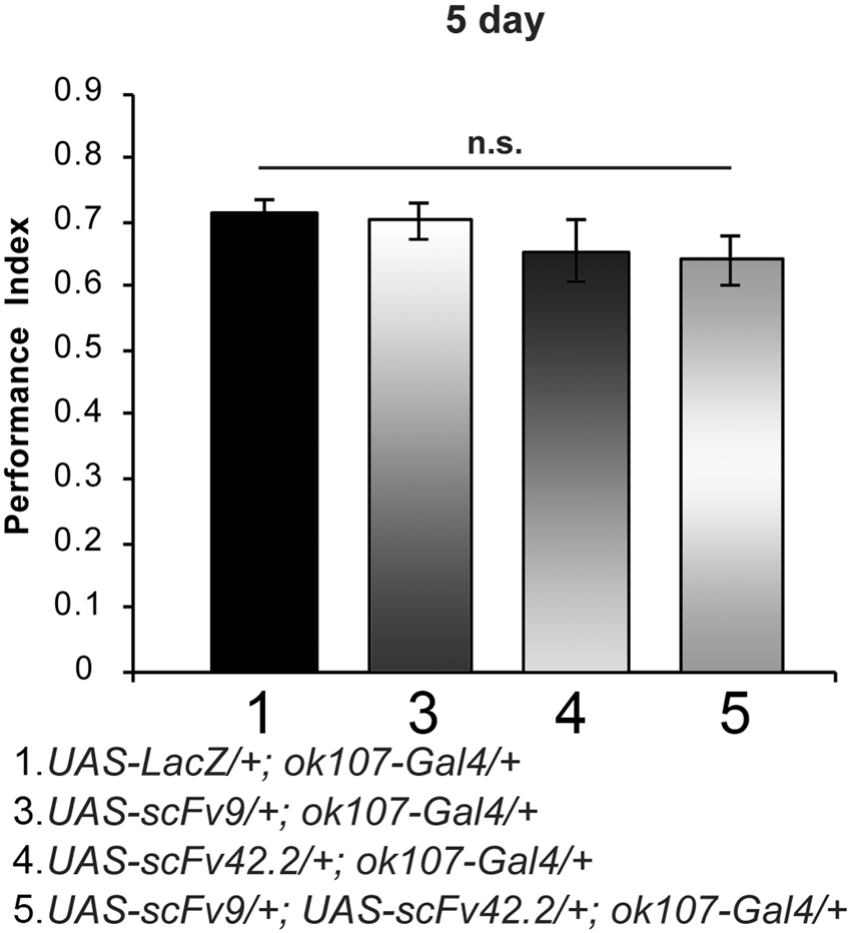
Exogenous expression of scFvs does not perturb memory acquisition. Flies were trained at day 5 post-eclosion using olfactory classical conditioning and tested immediately after training. Memory performance index is shown for control flies (*UAS-LacZ*/+; *ok107-Gal4*/+), flies expressing scFv9 alone (*UAS-scFv9*/+; *ok107-Gal4*/+), flies expressing scFv42.2 alone (*UAS-scFv42*.*2*/+; *ok107-Gal4*/+), and flies co-expressing scFv9 and scFv42.2 (*UAS-scFv9*/+; *UAS-scFv42*.*2*/+; *ok107-Gal4*/+). Five-day-old flies expressing scFv9, scFv42.2, or both perform at equivalent levels than control flies (*p* = 0.9999, *p* = 0.9989, and *p* = 0.9884, respectively). Error bars indicate SEM; n = 10 per group; n.s. (not significant).

Before testing the protective effects of two known anti-Aβ42 scFv antibodies^12^ on Aβ42 in the memory paradigm, we examined whether expression of the scFvs, alone or in combination, altered memory parameters in the absence of Aβ42. Expression of scFv9 alone, scFv42.2 alone, or both scFvs combined results in no significant differences in memory performance compared with that of control flies (Fig. 2; *p* > 0.9999, *p* > 0.9989, and *p* > 0.9884, respectively). In flies expressing scFv9 alone, scFv42.2 alone, or both combined avoidance to electric shock is similar to that of control flies expressing LacZ (Table 4; *p* = 0.9999, *p* = 0.9943, an *p* = 9961, respectively). Similarly, in flies expressing scFv9 alone, scFv42.2 alone, or both combined avoidance to octanol is comparable to that of control flies expressing LacZ (Table 5; *p* = 0.9999, *p* = 0.5803, and *p* = 6295, respective). Finally, flies expressing scFv9 alone, scFv42.2 alone, or both combined avoidance to benzaldehyde is similar to that of control flies expressing LacZ (Table 6; *p* = 0.9999, *p* = 0.9999, and *p* = 0.9999, respectively). Overall, expression of scFvs in MB neurons cause no disturbances in memory acquisition or in stimuli perception, and thus can be used to determine their ability to protect against Aβ42-mediated memory loss.

**Table 4:** Shock avoidance of flies expressing scFvs alone at day 5.

Genotype	90 V Shock Avoidance	*p-value*
*1-UAS-LacZ*/+; *ok107-Gal4*/+	0.6684 ± 0.0619	—
*3-UAS-svFv9*/+; *ok107-Gal4*/+	0.7050 ± 0.0384	*0.9999*
*4-UAS-svFv42.2*/+; *ok107-Gal4*/+	0.5904 ± 0.0542	*0.9943*
*5-UAS-svFv9*/+; *UAS-scFv42.2*/+; *ok107-Gal4*/+	0.6033 ± 0.0396	*0.9961*

Avoidance to an electric shock of 90 V for each corresponding genotype and their *p*-value for statistical significance versus the control (#1).

**Table 5:** Odor avoidance for 3-octanol of flies expressing scFvs alone at day 5.

Genotype	Octanol Avoidance	*p-value*
1	0.6018 ± 0.0690	—
3	0.6439 ± 0.0929	*0*.*9999*
4	0.4022 ± 0.0848	*0*.*5803*
5	0.3952 ± 0.0686	*0*.*6295*

Avoidance to the odor 3-octanol for each corresponding genotype and their *p*-value for statistical significance versus the control (#1). Complete genotypes: 1-*UAS-LacZ*/+; *ok107-Gal4*/+, 3-*UAS-scFv9*/+; *ok107-Gal4*/+, 4-*UAS-scFv42*.*2*/+; *ok107-Gal4*/+ and 5-*UAS-scFv9*/+; *UAS-scFv42*.*2*/+; *ok107-Gal4*/+.

**Table 6:** Odor avoidance for benzaldehyde of flies expressing scFvs alone at day 5.

Genotype	Benzaldehyde Avoidance	*p-value*
1	0.4988 ± 0.0844	—
3	0.4426 ± 0.0235	*0*.*9999*
4	0.4716 ± 0.1274	*0*.*9999*
5	0.4717 ± 0.1075	*0*.*9999*

Avoidance to the odor benzaldehyde for each corresponding genotype and their *p*-value for statistical significance versus the control (#1). Complete genotypes: 1-*UAS-LacZ*/+; *ok107-Gal4*/+, 3-*UAS-scFv9*/+; *ok107-Gal4*/+, 4-*UAS-scFv42*.*2*/+; *ok107-Gal4*/+ and 5-*UAS-scFv9*/+; *UAS-scFv42*.*2*/+; *ok107-Gal4*/+.

### scFv9 suppresses Aβ42-mediated memory deficits in *Drosophila*

**Figure 3:**
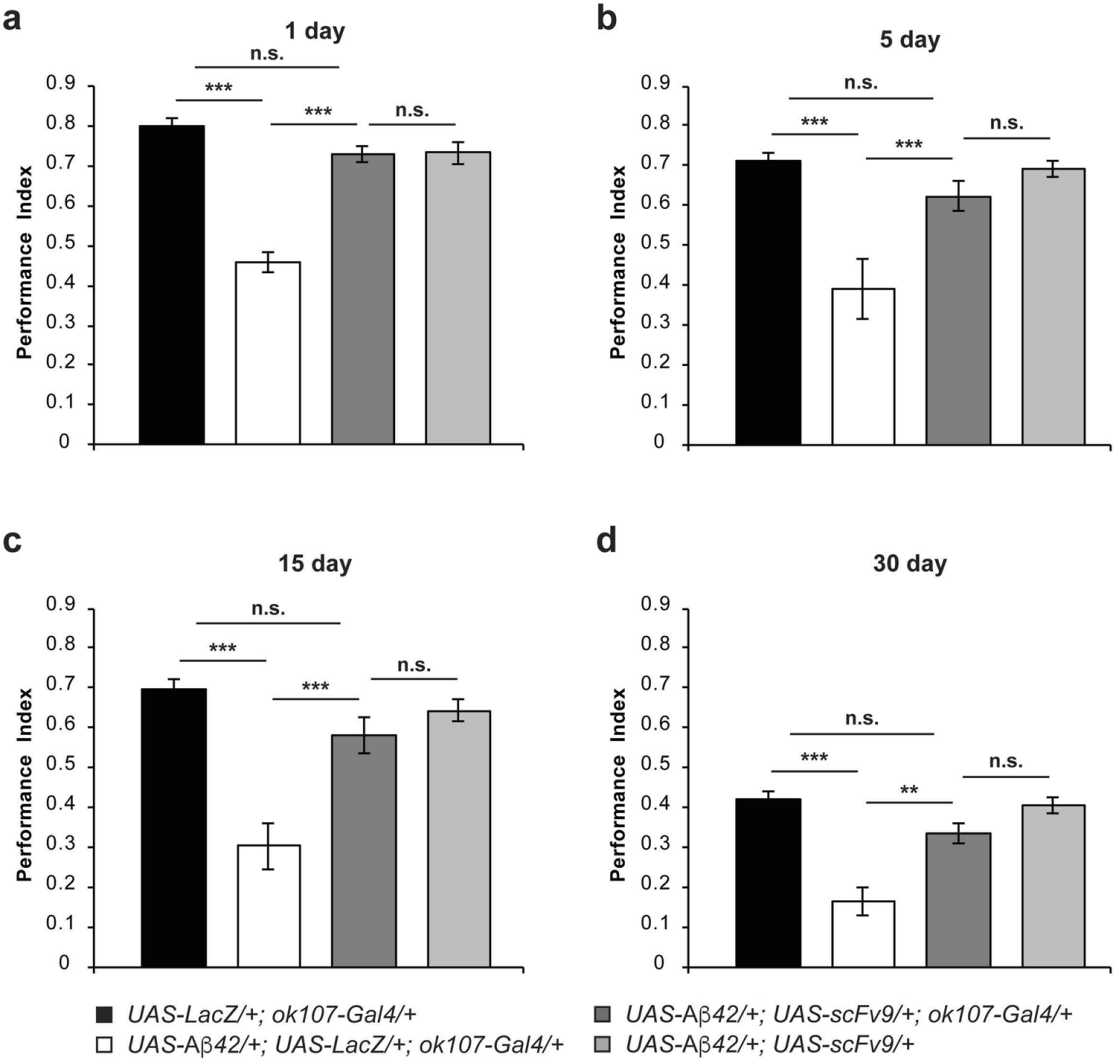
ScFv9 suppresses memory impairment in *Drosophila* expressing Aβ42. Flies were trained at days 1 (**a**), 5 (**b**), 15 (**c**), or 30 (**d**) post-eclosion using olfactory classical conditioning and tested immediately after training. Memory performance index is shown for control flies (*UAS-LacZ*/+; *ok107-Gal4*/+), flies expressing Aβ42 (*UAS-Aβ42*/+; *UAS-LacZ*/+; *ok107-Gal4*/+), flies co-expressing Aβ42 and scFv9 (*UAS-Aβ42*/*UAS-scFv9*; *ok107-Gal4*/+), and the corresponding control flies not carrying the Gal4 driver (*UAS-Aβ42*/*UAS-scFv9*). (**a**) One-day-old flies expressing Aβ42 and LacZ in the MB neurons display a significantly lower memory performance (*p* < 0.0001) than control flies expressing LacZ alone. Flies co-expressing scFv9 and Aβ42 performed at a significantly higher level than flies co-expressing Aβ42 and LacZ (*p* < 0.0001), but performed similar to control flies (*UAS-LacZ*/+; *ok107-Gal4*/+, *p* = 0.1997; *UAS-Aβ42*/*UAS-scFv9*, *p* = 0.8392). (**b**) Five-day-old flies expressing Aβ42 and LacZ in the MB neurons display a significantly lower memory performance (*p* = 0.0001) than control flies expressing LacZ alone. Flies co-expressing scFv9 and Aβ42 performed at a significantly higher level than flies co-expressing Aβ42 and LacZ (*p* = 0.0058), but performed similar to control flies (*UAS-LacZ*/+; *ok107-Gal4*/+, *p* = 0.5994; *UAS-Aβ42*/*UAS-scFv9*, *p* = 0.7294). (**c**) Fifteen-day-old flies expressing Aβ42 and LacZ in the MB neurons display a significantly lower memory performance (*p* < 0.0001) than control flies expressing LacZ alone. Flies co-expressing scFv9 and Aβ42 performed at a significantly higher level than flies co-expressing Aβ42 and LacZ (*p* = 0.0003), but performed similar to control flies (*UAS-LacZ*/+; *ok107-Gal4*/+, *p* = 0.1901; *UAS-Aβ42*/*UAS-scFv9*, *p* = 0.2954). (**d**) Thirty-day-old flies expressing Aβ42 and LacZ in the MB neurons display a significantly lower memory performance (*p* = 0.0001) than control flies expressing LacZ alone. Flies co-expressing scFv9 and Aβ42 performed at a significantly higher level than flies co-expressing Aβ42 and LacZ (*p* = 0.0126), but performed similar to control flies (*UAS-LacZ*/+; *ok107-Gal4*/+, *p* = 0.3410;+/+; *UAS-Aβ42*/*UAS-scFv9*, *p* = 0.5029). Error bars indicate SEM; n = 10 per group; **p < 0.01, ***p < 0.001; n.s. (not significant).

Once we established that scFvs have no deleterious effects on memory, we assessed their ability to protect flies against the memory deficits triggered by Aβ42. First, we describe the consequences of co-expressing scFv9 targeting the N-terminal region of Aβ42 (Aβ1-16). Flies co-expressing scFv9 and Aβ42 in MB neurons (*UAS-Aβ42*/*UAS-scFv9*; *ok107-Gal4*/+) perform at a significantly higher level than flies co-expressing Aβ42 and LacZ (*UAS-Aβ42*/+; *UAS-LacZ*/+; *ok107-Gal4*/+) at all ages tested (Fig. 3; d1: *p* < 0.0001; d5: *p* = 0.0058; d15: *p* = 0.0003; d30: *p* = 0.0126). Furthermore, memory performance of one- (Fig. 3a), five- (Fig. 3b), fifteen- (Fig. 3c), and thirty-day-old (Fig. 3d) flies co-expressing scFv9 and Aβ42 is statistically undistinguishable from the corresponding control groups: control flies expressing LacZ (*UAS-LacZ*/+; *ok107-Gal4*/+) and flies bearing the *scFv9* and *Aβ* *42* transgenes without Gal4 driver (*UAS-Aβ*42/*UAS-scFv9*). Additionally, avoidance of flies co-expressing scFv9 and Aβ42 and control flies bearing the *scFv9* and *Aβ* *42* transgenes without Gal4 driver to electric shock (Table 7), octanol (Table 8), and benzaldehyde (Table 9) are equivalent to that of control flies expressing LacZ alone. Thus, indicating that scFv9 and its combination with Aβ42 has no effect in stimuli perception. Therefore, the scFv9 antibody restores memory performance at all ages tested, including thirty-day-old flies for which Aβ42 induces an abrupt memory loss. These results reveal a highly protective effect of the scFv9 antibody targeting the N-terminal region of Aβ42.

**Table 7:** Shock avoidance of flies co-expressing scFvs and Aβ42.

Genotype	90 V Shock Avoidance	*p-value*
*1-UAS-LacZ*/+; *ok107-Gal4*/+	0.6744 ± 0.0513	—
*2-UAS-Aβ42*/+; *UAS-LacZ*/+; *ok107-Gal4*/+	0.7611 ± 0.0368	*0*.*9106*
*6-UAS-Aβ42*/*UAS-svFv9*; *ok107-Gal4*/+	0.6795 ± 0.0274	*0*.*9999*
*7-UAS-Aβ42*/+; *UAS-svFv42.2*/+; *ok107-Gal4*/+	0.8149 ± 0.0201	*0*.*3698*
*8-UAS-Aβ42/UAS-svFv9*; *UAS-scFv42.2*/+; *ok107-Gal4*/+	0.7287 ± 0.0438	*0*.*9929*
*9-UAS-Aβ42/UAS-svFv9*	0.6518 ± 0.0416	*0*.*9999*
*10-UAS-Aβ42*/+; *UAS-svFv42.2*/+	0.6723 ± 0.0415	*0*.*9999*
*11-UAS-Aβ42/UAS-svFv9; UAS-scFv42.2*/+	0.6473 ± 0.0507	*0*.*9997*

Avoidance to an electric shock of 90 V for each corresponding genotype and their *p*-value for statistical significance versus the control (#1).

**Table 8:** Odor avoidance for 3-octanol of flies co-expressing scFvs and Aβ42.

Genotype	Octanol Avoidance	*p-value*
1	0.8116 ± 0.0121	—
2	0.5096 ± 0.1088	*0*.*0616*
6	0.7191 ± 0.0316	*0*.*9775*
7	0.4036 ± 0.1342	*0*.*0120*
8	0.6562 ± 0.1338	*0*.*8329*
9	0.7179 ± 0.0427	*0*.*9758*
10	0.6947 ± 0.0512	*0*.*9235*
11	0.6987 ± 0.0423	*0*.*9354*

Avoidance to the odor 3-octanol for each corresponding genotype and their *p*-value for statistical significance versus the control (1). Complete genotypes: 1-*UAS-LacZ*/+; *ok107-Gal4*/+, 2-*UAS-Aβ42*/+; *UAS-LacZ*/+; *ok107-Gal4*/+, 6-*UAS-Aβ42*/*UAS-svFv9*; *ok107-Gal4*/+, 7-*UAS-Aβ42*/+; *UAS-svFv42*.*2*/+; *ok107-Gal4*/+ and 8-*UAS-Aβ42*/*UAS-svFv9*; *UAS-scFv42*.*2*/+; *ok107-Gal4*/+, 9-*UAS-Aβ42*/+; *UAS-scFv9*/+, 10-*UAS-Aβ42*/+; *UAS-scFv42*.*2*/+, *and* 11-*UAS-Aβ42*/*UAS-scFv9*; *UAS-scFv42*.*2*/+.

**Table 9:** Odor avoidance for benzaldehyde of flies co-expressing scFvs and Aβ42.

Genotype	Benzaldehyde Avoidance	*p-value*
1	0.3944 ± 0.0805	—
2	0.1420 ± 0.1144	*0*.*5339*
6	0.4061 ± 0.0921	*0*.*9999*
7	0.4247 ± 0.0741	*0*.*9999*
8	0.4195 ± 0.1087	*0*.*9999*
9	0.4530 ± 0.0665	*0*.*9998*
10	0.4216 ± 0.0896	*0*.*9999*
11	0.3505 ± 0.1162	*0*.*9999*

Avoidance to the odor benzaldehyde for each corresponding genotype and their *p*-value for statistical significance versus the control (1). Complete genotypes: 1-*UAS-LacZ*/+; *ok107-Gal4*/+, 2-*UAS-Aβ42*/+; *UAS-LacZ*/+; *ok107-Gal4*/+, 6-*UAS-Aβ42*/*UAS-svFv9*; *ok107-Gal4*/+, 7-*UAS-Aβ42*/+; *UAS-svFv42*.*2*/+; *ok107-Gal4*/+ and 8-*UAS-Aβ42*/*UAS-svFv9*; *UAS-scFv42*.*2*/+; *ok107-Gal4*/+, 9-*UAS-Aβ42*/+; *UAS-scFv9*/+, 10-*UAS-Aβ42*/+; *UAS-scFv42*.*2*/+, *and* 11-*UAS-Aβ42*/*UAS-scFv9*; *UAS-scFv42*.*2*/+.

### scFv42.2 partially suppresses Aβ42-mediated memory deficits in *Drosophila*

**Figure 4:**
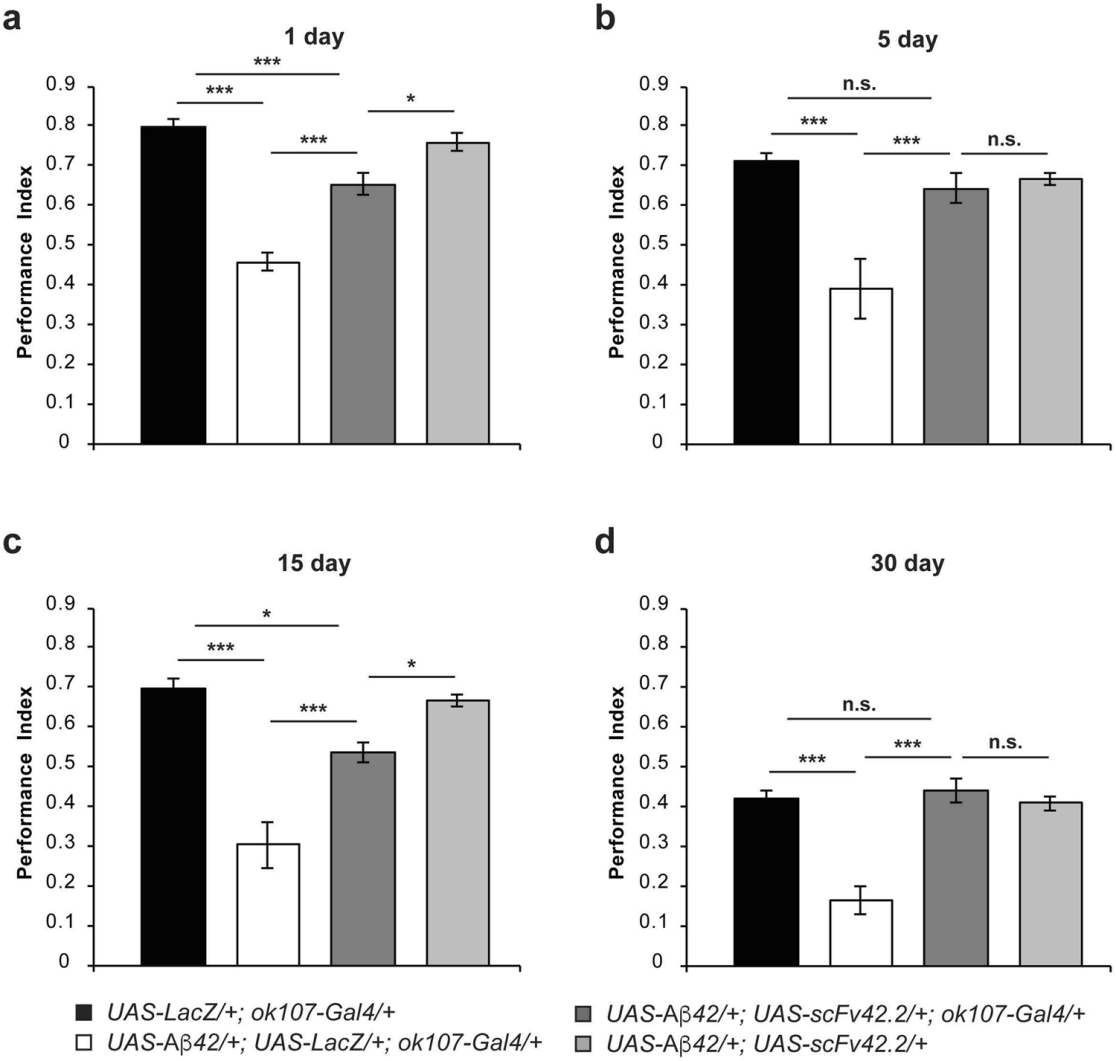
ScFv42.2 suppresses memory impairment in *Drosophila* expressing Aβ42. Flies were trained at days 1 (**a**), 5 (**b**), 15 (**c**), or 30 (**d**) post-eclosion using olfactory classical conditioning and tested immediately after training. Memory performance index is shown for control flies (*UAS-LacZ*/+; *ok107-Gal4*/+), flies expressing Aβ42 (*UAS-Aβ42*/+; *UAS-LacZ*/+; *ok107-Gal4*/+), flies co-expressing Aβ42 and scFv42.2 (*UAS-Aβ42*/+; *UAS-scFv42*.*2*/+; *ok107-Gal4*/+), and the corresponding control flies not carrying the Gal4 driver (*UAS-Aβ42*/+; *UAS-scFv42*.*2*/+). (**a**) One-day-old flies expressing Aβ42 and LacZ in MB neurons display a significantly lower memory performance (*p* < 0.0001) than control flies expressing LacZ alone. Flies co-expressing scFv42.2 and Aβ42 performed at a significantly higher level than flies co-expressing Aβ42 and LacZ (*p* = 0.0002), but slightly lower than control flies (*UAS-LacZ*/+; *ok107-Gal4*/+, *p* = 0.0019; *UAS-Aβ42*/+; *UAS-scFv42*.*2*/+, *p* = 0.0092). (**b**) Five-day-old flies expressing Aβ42 and LacZ in the MB neurons display a significantly lower memory performance (*p* = 0.0001) than control flies expressing LacZ alone. Flies co-expressing scFv42.2 and Aβ42 performed at a significantly higher level than flies co-expressing Aβ42 and LacZ (*p* = 0.0027), but performed similar to control flies (*UAS-LacZ*/+; *ok107-Gal4*/+, *p* = 0.7808; *UAS-Aβ42*/+; *UAS-scFv42*.*2*/+, *p* = 0.9429). (**c**) Fifteen-day-old flies expressing Aβ42 and LacZ in the MB neurons display a significantly lower memory performance (*p* < 0.0001) than control flies expressing LacZ alone. Flies co-expressing scFv42.2 and Aβ42 performed at a significantly higher level than flies co-expressing Aβ42 and LacZ (*p* = 0.0039), but slightly lower than control flies (*UAS-LacZ*/+; *ok107-Gal4*/+, *p* = 0.0459; *UAS-Aβ42*/+; *UAS-scFv42*.*2*/+, *p* = 0.0392). (**d**) Thirty-day-old flies expressing Aβ42 and LacZ in the MB neurons display a significantly lower memory performance (*p* = 0.0001) than control flies expressing LacZ alone. Flies co-expressing the scFv42.2 and Aβ42 performed at a significantly higher level than flies co-expressing the Aβ42 and LacZ (*p* < 0.0001), but performed similar to control flies (*UAS-LacZ*/+; *ok107-Gal4*/+, *p* = 0.9945; *UAS-Aβ42*/+; *UAS-scFv42*.*2*/+, *p* = 0.9996). Error bars indicate SEM; n = 10 per group; *p < 0.05, ***p < 0.001; n.s. (not significant).

We next tested the anti-Aβ42 scFv antibody targeting the C-terminal region of Aβ42 (Aβx-42), scFv42-2. Flies co-expressing scFv42.2 and Aβ42 (*UAS-Aβ*42/+; *UAS-scFv*42.*2*/+; *ok107-Gal4*/+) perform at a significantly higher level than flies expressing Aβ42 and LacZ (*UAS-Aβ42*/+; *UAS-LacZ*/+; *ok107-Gal4*/+) at each time point (Fig. 4; d1: *p* = 0.0002; d5: *p* = 0.0027; d15: *p* = 0.0039; d30: *p* < 0.0001). Furthermore, memory performance of one- (Fig. 4a) and fifteen-day-old (Fig. 4c) flies co-expressing scFv42.2 and Aβ42 show partial rescue since their memory values are significantly lower than those of the two corresponding control groups (*UAS-LacZ*/+; *ok107-Gal4*/+; d1: *p* = 0.0019, and d15: *p* = 0.0419; and *UAS-Aβ42*/+; *UAS-scFv42*.*2*/+; d1: *p* = 0.0092 and d15 *p* = 0.0392). Remarkably, five- (Fig. 4b) and thirty-day-old (Fig. 4d) flies co-expressing Aβ42 and scFv42.2 perform at the same level as the corresponding control groups. Moreover, avoidance of flies co-expressing scFv42.2 and Aβ42 to electric shock (Table 7) and benzaldehyde (Table 9) are statistically undistinguishable from those of control flies. However, avoidance to octanol is significantly lower than that in control flies (Table 8), which makes the memory rescue even more notable and explains, in part, the lower memory scores at days 1 and 15. Additionally, avoidance to electric shock and odors in control flies bearing the *scFv42*.*2* and *Aβ42* transgenes without Gal4 driver are equivalent to that of control flies (Tables 7–9). Despite the lower protective effect for scFv42.2 at days 1 and 15, the performance levels of both anti-Aβ42 scFv antibodies are not significantly different from each other (Tables 10–13). Together, these results reveal a potent protective effect of the scFv42.2 against the memory impairments associated with Aβ42 neurotoxicity.

**Table 10:** Memory performance between paired genotypes at day 1 (*p* values).

Genotypes	2	6	7	8
1	<*0.0001*	*0.1997*	*0.0019*	*0.1477*
2	—	<*0.0001*	*0.0002*	<*0.0001*
6	—	—	*0.2630*	*0.9998*
7	—	—	—	*0.3404*

Complete genotypes: 1-*UAS-LacZ*/+; *ok107-Gal4*/+, 2-*UAS-Aβ42*/+; *UAS-LacZ*/+; *ok107-Gal4*/+, 6-*UAS-Aβ42*/*UAS-svFv9*; *ok107-Gal4*/+, 7-*UAS-Aβ42*/+; *UAS-svFv42*.*2*/+; *ok107-Gal4*/+ and 8-*UAS-Aβ42*/*UAS-scFv9*; *UAS-scFv42*.*2*/+; *ok107-Gal4*/+.

**Table 11:** Memory performance between paired genotypes at day 5 (*p* values).

Genotypes	2	6	7	8
1	*0.0001*	*0.5994*	*0.7808*	*0.8445*
2	—	*0.0058*	*0.0027*	*0.0019*
6	—	—	*0.9979*	*0.9920*
7	—	—	—	>*0.9999*

Complete genotypes: 1-*UAS-LacZ*/+; *ok107-Gal4*/+, 2-*UAS-Aβ42*/+; *UAS-LacZ*/+; *ok107-Gal4*/+, 6-*UAS-Aβ42*/*UAS-svFv9*; *ok107-Gal4*/+, 7-*UAS-Aβ42*/+; *UAS-svFv42*.*2*/+; *ok107-Gal4*/+and 8-*UAS-Aβ42*/*UAS-svFv9*; *UAS-scFv42*.*2*/+; *ok107-Gal4*/+.

**Table 12:** Memory performance between paired genotypes at day 15 (*p* values).

Genotypes	2	6	7	8
1	0.0001	0.1901	0.0459	0.0062
2	—	0.0003	0.0039	0.0127
6	—	—	0.9287	0.5627
7	—	—	—	0.9657

Complete genotypes: 1-*UAS-LacZ*/+; *ok107-Gal4*/+, 2-*UAS-Aβ42*/+; *UAS-LacZ*/+; *ok107-Gal4*/+, 6-*UAS-Aβ42*/*UAS-svFv9*; *ok107-Gal4*/+, 7-*UAS-Aβ42*/+; *UAS-svFv42*.*2*/+; *ok107-Gal4*/+ and 8-*UAS-Aβ42*/*UAS-svFv9*; *UAS-scFv42*.*2*/+; *ok107-Gal4*/+.

**Table 13:** Memory performance between paired genotypes at day 30 (*p* values)

Genotypes	2	6	7	8
1	*0.0001*	*0.3410*	*0.9945*	*0.9996*
2	—	*0.0126*	<*0.0001*	*0.0002*
6	—	—	*0.1791*	*0.4490*
7	—	—	—	*0.9745*

Complete genotypes: 1-*UAS-LacZ*/+; *ok107-Gal4*/+, 2-*UAS-Aβ42*/+; *UAS-LacZ*/+; *ok107-Gal4*/+, 6-*UAS-Aβ42*/*UAS-svFv9*; *ok107-Gal4*/+, 7-*UAS-Aβ42*/+; *UAS-svFv42*.*2*/+; *ok107-Gal4*/+and 8-*UAS-Aβ42*/*UAS-svFv9*; *UAS-scFv42*.*2*/+; *ok107-Gal4*/+.

### Combined scFv9 and scFv42.2 suppress Aβ42-mediated memory deficits in *Drosophila*

**Figure 5:**
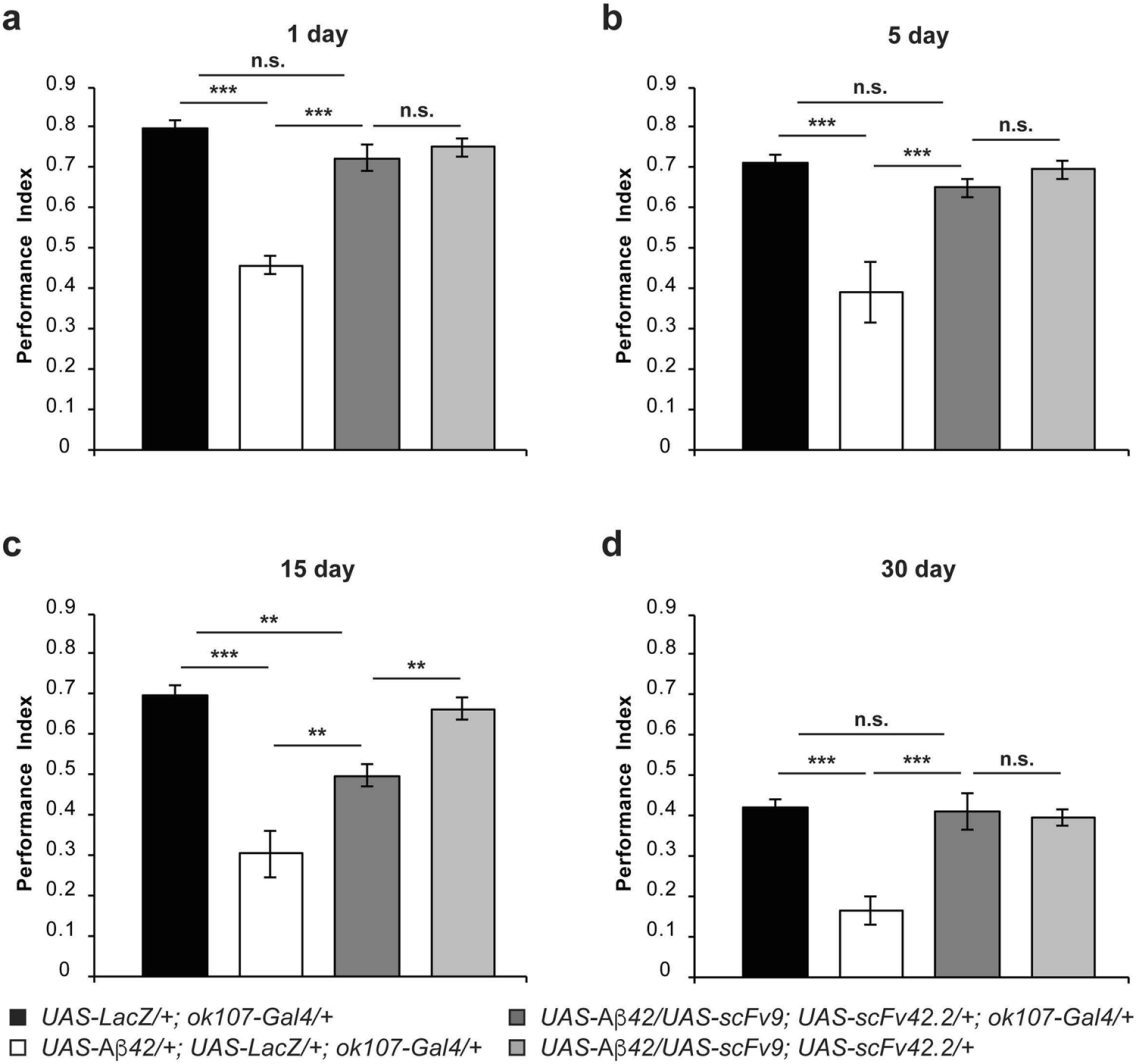
Combined expression of scFv9 and scFv42.2 suppresses memory impairment in *Drosophila* expressing Aβ42. Flies were trained at days 1 (**a**), 5 (**b**), 15 (**c**), or 30 (**d**) post-eclosion using olfactory classical conditioning and tested immediately after training. Memory performance index is shown for control flies (*UAS-LacZ*/+; *ok107-Gal4*/+), flies expressing Aβ42 (*UAS-Aβ42*/+; *UAS-LacZ*/+; *ok107-Gal4*/+), flies co-expressing Aβ42, scFv42.2 and scFv9 (*UAS-Aβ42*/*UAS-scFv9*; *UAS-scFv42*.*2*/+; *ok107-Gal4*/+) and the corresponding control flies not carrying the Gal4 driver (*UAS-Aβ42*/*UAS-scFv9*/+; *UAS-scFv42*.*2*/+). (**a**) One-day-old flies expressing Aβ42 and LacZ in the MB neurons display a significantly lower memory performance (*p* < 0.0001) than control flies expressing LacZ alone. Flies co-expressing scFv9 and scFv42.2 in combination with Aβ42 performed at a significantly higher level than flies co-expressing Aβ42 and LacZ (*p* < 0.0001), but performed similar to control flies (*UAS-LacZ*/+; *ok107-Gal4*/+, *p* = 0.1477; *UAS-Aβ42*/*UAS-scFv9*; *UAS-scFv42*.*2*/+, *p* = 0.2318). (**b**) Five-day-old flies expressing Aβ42 and LacZ in the MB neurons display a significantly lower memory performance (*p* = 0.0001) than control flies expressing LacZ alone. Flies co-expressing scFv9 and scFv42.2 in combination with Aβ42 performed at a significantly higher level than flies co-expressing Aβ42 and LacZ (*p* = 0.0019), but performed similar to control flies (*UAS-LacZ*/+; *ok107-Gal4*/+, *p* = 0.8445; *UAS-Aβ42*/*UAS-scFv9*; *UAS-scFv42*.*2*/+, *p* = 0.9284). (**c**) Fifteen-day-old flies expressing Aβ42 and LacZ in the MB neurons display a significantly lower memory performance (*p* < 0.0001) than control flies expressing LacZ alone. Flies co-expressing scFv9 and scFv42.2 in combination with Aβ42 performed at a significantly higher level than flies co-expressing Aβ42 and LacZ (*p* = 0.0127), but slightly lower than control flies (*UAS-LacZ*/+; *ok107-Gal4*/+, *p* = 0.0062; *UAS-Aβ42*/*UAS-scFv9*; *UAS-scFv42*.*2*/+, *p* = 0.0023). (**d**) Thirty-day-old flies expressing Aβ42 and LacZ in the MB neurons display a significantly lower memory performance (*p* = 0.0001) than control flies expressing LacZ alone. Flies co-expressing scFv9 and scFv42.2 in combination with Aβ42 performed at a significantly higher level than flies co-expressing Aβ42 and LacZ (*p* = 0.0002), but performed similar to control flies (*UAS-LacZ*/+; *ok107-Gal4*/+, *p* = 0.9996; *UAS-Aβ42*/*UAS-scFv9*; *UAS-scFv42*.*2*/+, *p* = 0.9999). Error bars indicate SEM; n = 10 per group; **p < 0.01, ***p < 0.001; n.s. (not significant).

We previously reported further protective activity when the two anti-Aβ42 scFvs were co-expressed in several *Drosophila* assays (eye morphology, dendritic architecture of MB neurons), but no interaction in other assays (climbing, neuronal cell death)^13^. To determine whether there is an interaction between the two scFvs in the memory paradigm, we next combined expression of both anti-Aβ42 scFvs and Aβ42 in MB neurons. Flies co-expressing both scFvs and Aβ42 (*UAS-*Aβ42/*UAS-scFv9*; *UAS-scFv42*.*2*/+; *ok107-Gal4*/+) perform at a significantly higher level than flies expressing Aβ42 and LacZ at all ages tested (Fig. 5; d1: *p* < 0.0001; d5: *p* = 0.0019; d15: *p* = 0.0127; d30: *p* = 0.0002). Furthermore, one- (Fig. 5a), five- (Fig. 5b), and thirty-day-old (Fig. 5d) flies co-expressing both antibodies and Aβ42 perform at the same statistical level than the corresponding control groups (*UAS-LacZ*/+; *ok107-Gal4*/+ and *UAS-*Aβ42/*UAS-scFv9*; *UAS-scFv42*.*2*/+). Moreover, the memory performance of flies with combined expression of both scFvs is statistically comparable to the performance of flies expressing only one scFv (Tables 10–13) at 1, 5, and 30 days of age. However, fifteen-day-old flies co-expressing the two scFvs and Aβ42 perform slightly lower than the corresponding control groups (Fig. 5c; *p* = 0.0062 and *p* = 0.0023). Finally, avoidance indexes of flies co-expressing both antibodies and Aβ42 and the corresponding control flies to electric shock (Table 5), octanol (Table 8), and benzaldehyde (Table 9) are similar to that of control flies expressing LacZ, indicating no perception deficits. Overall, these results indicate that combined expression of these scFvs do not trigger an additive effect on memory performance.

## Discussion

Over the last 20 years, we have witnessed remarkable advances in the understanding of the molecular mechanisms mediating AD pathogenesis. Unfortunately, these advances have not resulted in therapies that can efficiently halt the progression of the disease. Despite recent setbacks, passive immunotherapy continues to be a highly promising therapeutic approach against AD^7–10^. ScFvs are engineered antibodies that can complement or substitute full antibodies due to their limited induction of the cellular immune response and improved brain penetration, which could compensate for their shorter half-life^11^. Here, we tested the neuroprotective activity of two anti-Aβ42 scFv antibodies, scFv9 and scFv42.2^12^, in a learning and memory paradigm in *Drosophila*. Expression of scFv9 triggered a consistent neuroprotective activity at all time points, whereas scFv42.2 showed robust memory recovery, except for partial recovery at days 1 and 15. The stronger performance of scFv9 is consistent with the exposure of the N-terminal region of Aβ42 in aggregated assemblies, which provides direct access of this antibody to all forms of Aβ42, from monomeric to fibrillar assemblies. However, the humanized N-terminal antibody bapineuzumab has not demonstrated functional protection, so far, and seems to be discarded for further clinical trials^10, 35^. In contrast, the central and C-terminal regions of Aβ42 are buried in the core of fibrillar Aβ42 conformations, making them inaccessible to antibodies like scFv42.2. Antibodies against the central region only bind Aβ42 monomers to promote their degradation or prevent their aggregation into toxic assemblies^12, 36^. Despite its limited access to Aβ42 monomers, scFv42.2 has also shown a strong neuroprotective activity in transgenic flies, which could be partially due to higher expression levels^13^. Solanezumab, a promising humanized antibody against the central domain of Aβ42, is the only antibody recognizing a lineal epitope that has demonstrated partial clinical benefits, so far^8, 37, 38^, and is still active in clinical trials. Thus, we need to further understand the protective mechanisms of monomer-specific anti-Aβ42 antibodies to exploit the cellular pathways mediating their activity.

Since our two scFv antibodies bind non-overlapping Aβ42 domains and seem to operate by different mechanisms, we suggested the possibility of beneficial interactions (cooperative or synergistic) when combining both scFvs. We previously observed benefits from co-expressing both scFvs in some assays, but not in others^13^. Here we show that co-expression of the scFv42.2 and scFv9 does not improve memory performance, partly because the memory levels in flies expressing scFv9 are already at the level of control flies. Cell-, tissue-, and assay-specific dynamics of Aβ42 aggregation and neurotoxicity may account for different access and/or affinity of each scFv to Aβ42 and, thus, explain the distinct effectiveness of the scFvs in each assay.

To date, large clinical trials employing anti-Aβ42 immunotherapy have produced disappointing results in early AD, MCI, and pre-symptomatic patients with plaques^7–10^. These negative results have raised strong dissent among experts regarding: (i) the amyloid hypothesis as the mechanism explaining AD pathogenesis and (ii) the role of Aβ42 as the main therapeutic target responsible for triggering other AD pathologies, including tau hyperphosphorylation. Despite the discouraging scenario emerging from the poor clinical results, these serious setbacks can be explained by the advanced brain degeneration in the selected patients and the low penetration of full antibodies into critical brain regions. Ongoing attempts to treat presymptomatic at-risk carriers of AD mutations, and improvements in the design of antibodies to avoid undesired effects and target conformational epitopes still provide hope for identifying the first disease-modifying therapy for AD^10, 39^. These continuing immunotherapy efforts are supported by strong preclinical results in animal models, which champion four non-exclusive hypotheses for the mechanisms mediating the benefits of Aβ42 immunotherapy. (1) The “peripheral sink” hypothesis posits that anti-Aβ42 antibodies sequester Aβ42 circulating in serum, which favors the diffusion of Aβ42 from the brain to blood vessels and, consequently, decreases the Aβ42 load in the brain^40^. The caveat to this model is that high levels of Aβ42 in cerebral blood vessels can increase the risk of vascular dementia (cerebral amyloid angiopathy) and may be responsible for amyloid-related imaging abnormalities (ARIA) observed in clinical trials with bapineuzumab and other related antibodies^5, 6^. (2) The second hypothesis proposes that the small amounts of anti-Aβ42 antibodies that penetrate the brain bind Aβ42 and promote Aβ42 clearance via microglia and macrophages^41^. (3) A third hypothesis suggests that anti-Aβ42 antibodies bind Aβ42 in relevant regions of the brain, preventing the aggregation of monomers or oligomers and/or promoting disaggregation of soluble and insoluble Aβ42 assemblies. Paradoxically, this mechanism would increase the amount of circulating soluble Aβ42 assemblies, which are proposed to be the most toxic Aβ42 species^42^. Finally, (4) the “Aβ42 masking” hypothesis proposes that anti-Aβ42 antibodies exert a neuroprotective activity by simply binding Aβ42 in the absence of adaptive immune response, Aβ42 degradation, or Aβ42 disaggregation^13, 43^. Under this scenario, the direct and stable binding of antibodies could promote Aβ42 aggregation into non-toxic conformations or mask (block) Aβ42 interactions with cellular substrates, thus suppressing Aβ42 neurotoxicity. In support of this masking hypothesis, several chaperones bind Aβ42 and promote aggregation into non-toxic assemblies under certain experimental conditions^43–45^. This hypothesis predicts that proteins or drugs that alter Aβ42 aggregation dynamics and interaction with cellular substrates will elicit neuroprotection without lowering the Aβ42 load. This mechanism would be equivalent to the sequestration of intracellular amyloids in the aggresome, which is proposed to store misfolded proteins bound to chaperones and other proteins in an organelle that prevents the mobility and toxicity of amyloids^46–48^. In the absence of such an organelle in the extracellular space, antibodies, secreted chaperones, and small molecules that can alter the pathogenic aggregation of Aβ42 should be highly beneficial while carrying low risks.

One of the main advantages of scFvs, camelids, and other engineered antibodies is their small size and relatively simple design, which facilitates their packaging in small viral vectors. These vectors can be used in the near future to directly target brain neurons, bypassing the problems associated with limited antibody diffusion across the blood-brain barrier. Although these technologies are far from clinical application, fast developments in gene therapy technologies may soon lead to targeted expression of anti-Aβ42 antibody fragments. The present work demonstrates the relevance of *Drosophila* for testing the protective activity of candidate genes and therapeutic agents in complex behavioral tasks, including the highly relevant learning and memory paradigm employed here. Our results sustain the feasibility of employing this assay in selective screenings of Aβ42-binding proteins, including scFv antibodies and drugs, before moving to costlier and time-consuming rodent models. These experiments can provide comparative data for proteins binding different Aβ42 regions or assemblies, and also determine the added value of combining several therapeutic agents.

## Experimental procedures

### Fly strains and genetics

Fly stocks were raised on standard cornmeal media at 25 °C. Flies carrying the UAS transgenes *w*; *UAS-Aβ42* ^29^, *w*; *UAS-scFv9* and *w*; *UAS-scFv42*.*2* ^13^ were previously described. The Gal4 line *ok107-Gal4* ^49^ was obtained from the Bloomington Stock Center at Indiana University (Bloomington, IN). Crosses between flies bearing the *ok107-Gal4* driver and the different UAS lines or a combination of them were set at 25 °C for 2 days and then transferred to 27 °C and 70% relative humidity on a 12 h light/dark cycle for development and aging until conditioning.

### Olfactory classical conditioning

Olfactory learning was assayed using olfactory classical conditioning procedures^50^. All behavioral experiments were performed under a dim red light at 26 °C and 80% relative humidity. Groups of 50–60 flies were transferred to small plastic tubes with a copper-grid floor to deliver the electric shock. A single cycle of training consisted of one presentation of (CS+) for 60 sec along with 90 V, 1.25 sec shock pulses (GRASS S48 Stimulator) every 5 sec, followed by the second odor presentation without associated shock (CS−) for another 60 sec. Odor presentations were separated by 30 sec of fresh air. For each N, two groups of flies of the same genotype were trained and tested simultaneously with the CS+ and CS− odors reversed. Benzaldehyde and 3-octanol were selected as the odor pairs.

After training, the animals were tested immediately in a runway in which they chose between avoiding the CS+ or the CS− odor. Performance index (PI) was calculated by subtracting the number of flies avoiding the CS− odor from the number of flies avoiding the CS+ odor, divided by the total number of flies, and averaged for the two reciprocal half experiments with reversed odors.

### Odor and shock acuity

Stimulus perception was evaluated by the preference to avoid the 90 V electric shock, octanol, or benzaldehyde naively before conditioning. Each stimulus was presented independently to flies of the different ages and the corresponding genotype. Odor and shock avoidance were calculated by subtracting the number of flies avoiding the odor or shock from the number of flies avoiding mineral oil (the solvent for the odors), divided by the total number of flies.

### Statistical analyses

Statistical analyses were performed using GraphPad Prism (v5.0c). All data presented represent the mean ± the standard error of the mean (SEM). The sample size was 10 for each group unless otherwise stated. As PI values are normally distributed^50–52^, parametric one-way ANOVA test followed by Tukey’s post-hoc or t-test comparisons were used for statistical analysis. Wilcoxon test was used to analyze significance from zero.

### Approvals/regulations

The experiments described were approved by the Environmental Health and Safety Committee of the University of Florida. All the methods described were carried out in accordance with the relevant guidelines and regulations.
